# Phytochemical composition, antioxidant, antibacterial, and enzyme inhibitory activities of organic extracts from flower buds of *Cleistocalyx operculatus* (Roxb.) Merr. et Perry

**DOI:** 10.5114/bta.2024.139753

**Published:** 2024-06-25

**Authors:** Doan Thien Thanh, Vo Kieu Oanh, Hoang Chinh Nguyen, Luong Thi My Ngan, Tran Trung Hieu

**Affiliations:** 1 Faculty of Biology and Biotechnology, VNUHCM-University of Science, Ho Chi Minh City, Vietnam; 2 Faculty of Applied Sciences, Ton Duc Thang University, Ho Chi Minh City, Vietnam; 3 School of Life and Environmental Sciences, Deakin University, Geelong, Australia

**Keywords:** *Cleistocalyx operculatus*, flower bud, phytochemical composition, antioxidant activity, antibacterial activity, enzyme inhibition

## Abstract

*Cleistocalyx operculatus* flower buds have been widely used in traditional medicine because of their rich content of bioactive constituents. In this study, we obtained seven solvent extracts from the flower buds and evaluated their total phenolic (TPC), flavonoid (TFC), tannin (TTC), triterpenoid saponin (TSC), and alkaloid (TAC) contents. We assessed antioxidant activities using the DPPH assay and also looked at antimicrobial and enzyme inhibitory effects. The water extract possessed the highest TPC (328.9 mg GAE/g extract), followed by ethanol, methanol, and hexane extracts (85.4–101.5 mg GAE/g extract). Chloroform, butanol, ethyl acetate, and ethanol extracts had high TSCs (245.4–287.2 mg OAE/g extract). The hexane extract was richest in TTC and TFC (32.7 mg CE/g extract and 81.1 mg QE/g extract, respectively). Ethanol and methanol extracts exhibited the strongest antioxidant activities (IC_50_ values of 25.2 and 30.3 μg/ml, respectively), followed by the water extract (IC_50_ of 40.2 μg/ml). The hexane extract displayed the most growth-inhibitory activity against *Helicobacter pylori* ATCC51932 and ATCC43504 strains and *Salmonella enterica* serovar *Typhimurium* ATCC13311 (MIC values of 0.06, 0.13, and 0.4 mg/ml, respectively). Moreover, the hexane extract revealed the strongest inhibition of *H. pylori* urease activity (IC_50_ of 4.51 μg/ml), whereas the water and methanol extracts had potent inhibitory effects on α-glucosidase activity (IC_50_ values of 9.9 and 15.1 μg/ml, respectively). These flower bud extracts could be used for health protection, especially in preventing bacterial infections and inhibiting enzymes associated with various human diseases. Further investigation into the application of *C. operculatus* flower buds in the food and pharmaceutical industries is necessary.

## Introduction

Growing global concerns about human health issues, such as infections caused by multiple drug-resistant bacteria and adverse effects of antibiotics and other synthetic drugs, have drawn public attention to the use of plant-derived natural products (Uysal et al., 2021). Particularly, phytochemicals such as alkaloids, phenolics, flavonoids, and saponins have received growing interest as bioactive sources for the food and pharmaceutical industries due to their properties in wound healing, antioxidant, antimicrobial, antidiabetic, and anticancer activities (Ye et al., 2013; Seo et al., 2014; Rebaya et al., 2015; Temesgen et al., 2022).

*Cleistocalyx operculatus* (Roxb.) Merr. et Perry (*Syzygium nervosum* D.C. or *Eugenia operculata* Roxb.) is an evergreen medicinal tree of the Myrtaceae family. The plant is found and cultivated in several tropical and subtropical countries such as Cambodia, China, Laos, India, Thailand, and Vietnam (Loi, 2001; Pengkumsri et al., 2019). In Vietnam, the tree is cultivated in many provinces in the Northern Delta and from the Midland to South-eastern areas for its leaves, flower buds, and wood. Its flower buds and leaves have been traditionally used in herbal tea and as ingredients in remedies to treat various disorders such as gastric inflammation, bacillary dysentery, diarrhea, colds, and fever (Loi, 2001; Ye et al., 2004; Wang et al., 2016; Pengkumsri et al., 2019; Pham et al., 2020). The decoction of the flower buds has also been used to treat influenza, indigestion, acne, itchy skin, and wound and skin infections (Loi, 2001; Pham et al., 2020). The leaves and bark are considered to have antiseptic properties, while the roots are known to have positive effects on jaundice and abdominal pain (Loi, 2001; Pham et al., 2020). The water extract of the flower buds was reported to contain the highest contents of phenolics and flavonoids and to possess potent antioxidant and antidiabetic effects (Mai et al., 2010). Flavonoid compounds isolated from the methanolic extract of the flower buds exhibited inhibitory activities against neuraminidase from influenza viruses (H1N1 and H9N2) (Dao et al., 2010), while cleistocaltones have been reported to display inhibitory effects on the respiratory syncytial virus (Song et al., 2019). The ethanolic extract of the flower buds was also found to have potent anti-inflammatory and antiosteoclastogenic effects (Tran et al., 2019a, 2019b), antioxidant and antibacterial properties against both Gram-positive and Gram-negative bacteria (Dung et al., 2008). A main flavonoid compound, 2′,4′-dihydroxy6′-methoxy-3′,5′-dimethylchalcone (DMC), isolated from the flower buds and seeds, was proven to serve as a chemotherapeutic agent for the prevention or treatment of lung, liver, pancreatic, and cervical cancers (Ye et al., 2004; Tuan et al., 2019; Utama et al., 2022) and inflammatory diseases (Tran et al., 2019a). Due to the safety and effective pharmacological activity of different solvent preparations from the flower buds of *C. operculatus*, the present study was conducted to obtain extracts from the flower buds using different solvents (hexane, chloroform, ethyl acetate, butanol, ethanol, methanol, and water) and evaluate the phytochemical content of these extracts (flavonoids, phenolics, tannins, triterpenoid saponins, and alkaloids), their antioxidant capacity, growth inhibitory activity against *Helicobacter pylori* and *Salmonella typhimurium*, and enzyme inhibitory effect on *H. pylori*-urease and α-glucosidase.

## Materials and methods

### Chemicals and reagents

Hexane, chloroform, ethyl acetate, butanol, ethanol, methanol, dimethyl sulfoxide (DMSO), atropine, newborn bovine serum (NBS), and bromocresol green (BCG) were purchased from Fisher Scientific (UK), while Folin–Ciocalteu was obtained from Merck (Darmstadt, Germany). Ascorbic acid, gallic acid, oleanolic acid, acarbose, catechin, quercetin, thiourea, 2,2-Diphenyl-1-picrylhydrazyl (DPPH), *p*-nitrophenyl-α-D-glucopyranoside (pNPG), and α-glucosidase from *S. cerevisiae* were obtained from Sigma-Aldrich (UK). Brain heart infusion broth (BHIB), Mueller Hinton II agar (MHA), Mueller Hinton II broth (MHB), and Brucella broth (BB) medium were purchased from HiMedia Laboratories LLC (Kennett Square, USA). Other chemicals were commercially available and of analytical purity.

### Plant material and extract preparation

The dried flower buds of *C. operculatus* (Roxb.) Merr. et Perry were obtained from the traditional medicine and herbal material market on Hai Thuong Lan Ong Street, Dist. 5^th^, Ho Chi Minh City, Vietnam, and identified at the Department of Plant Biotechnology and Biotransformation, Faculty of Biology and Biotechnology, VNUHCM-University of Science, Ho Chi Minh City. A voucher specimen was deposited in the Department’s herbarium under the code COFB1001.

The dried flower buds were ground to a powder and extracted with different solvents such as hexane, chloroform, ethyl acetate, butanol, ethanol, methanol, and distilled water. For each solvent type, 200 g of powder was extracted twice, each with 2 l of solvent for 24 h at room temperature. The liquid extracts were filtered and evaporated at 45°C using a rotary evaporator (Heidolph Instruments, Hei-VAP core) until completely dried.

### Bacterial strains and culture conditions

Two strains of *H. pylori*, ATCC 51932 and ATCC 43504, and *Salmonella enterica* serovar *Typhimurium* (*S. typhimurium*) ATCC 13311 were used to identify the antibacterial activity of the solvent extracts of *C. operculatus* flower buds. These bacterial strains were stored in a liquid nitrogen container until use. *H. pylori* was grown in BB medium with 10% NBS at 37°C for 48 h under microaerophilic conditions created by a 2.5 l Oxoid AnaeroJar and Oxoid CampyGen sachet (Thermo Fisher Scientific, Waltham, MA, USA). *S. typhimurium* was cultured in MHB medium at 37°C for 24 h.

### Determination of total phytochemical content in the flower bud extracts

The total phenolic content (TPC) in the extracts was quantified using the Folin–Ciocalteu assay (Wijekoon et al., 2011). Each dried flower bud extract of *C. operculatus* was dissolved in its solvent to a concentration of 1 mg/ml. Briefly, 450 μl of each extract was mixed with 2.25 ml of Folin–Ciocalteu reagent (10%) and incubated for 5 min. Then 1.8 ml of Na_2_CO_3_ (7%) was added and well mixed. After incubation for 30 min in the dark, the absorbance was measured at 765 nm using a spectrophotometer (UV-5100, Metash, Shanghai, China). The blank was prepared by using absolute ethanol to replace the extract. Gallic acid (0–100 μg/ml) was used to establish a standard curve (*y* = 0.0095*x* + 0.0029, *R*^2^ = 0.9978). The result was expressed as mg gallic acid equivalent (GAE)/g of dry extract.

The total flavonoid content (TFC) was quantified using the aluminum chloride colorimetric method (Wijekoon et al. 2011). Briefly, a mixture of 0.5 ml of each extract, 2.5 ml of distilled water, and 0.15 ml of sodium nitrite solution (5%) was prepared and incubated for 5 min. Then 0.3 ml of AlCl_3_ (5%) and 1 ml of NaOH 1 M were added. After incubating for 6 min, the absorbance of the mixture and the blank were measured at 510 nm. Quercetin was used to establish a standard curve (*y* = 0.0077*x* + 0.0124, *R*^2^ = 0.9991). The result was expressed as mg of quercetin equivalent (QE)/g of dry extract.

The total tannin content (TTC) was determined using the vanillin method (Broadhurst and Jones, 1978). Shortly, a mixture of 0.5 ml of each extract, 3 ml of vanillin (4% in methanol, w/v), and 1.5 ml of HCl was prepared. After incubation for 15 min in the dark, the absorbance of the mixture and the blank were measured at 500 nm. Catechin was used to establish a standard curve (*y =* 0.0012*x* + 0.0261, *R*^2^ = 0.9985). The result was shown as mg of catechin equivalent (CE)/g of dry extract.

The total triterpenoid saponin content (TSC) was also quantified using the vanillin method (Elouafy et al., 2023). Briefly, 1 ml of each extract was mixed with 0.2 ml of vanillin solution (5% in acetic acid) and 0.8 ml of perchloric acid 70%. After heating at 60°C for 15 min, the mixture was cooled down in an ice bath for 5 min and subsequently added with 5 ml of absolute acetic acid. The absorbance was measured at 544 nm. Oleanolic acid was used to establish a standard curve (*y* = 0.0021*x* + 0.0196, *R*^2^*=* 0.9955). The result was expressed as mg oleanolic acid equivalent (OAE)/g of dry extract.

The total alkaloid content (TAC) was determined using the BCG method (Fazel et al., 2008). Briefly, 1 ml of each extract was transferred to a separating funnel containing 5 ml of BCG solution (0.01%) and 5 ml of phosphate buffer solution (pH 4.7). The mixture was gently shaken and then extracted twice, each with 5 ml of chloroform. The chloroform extracts were collected and diluted in a 10 ml volumetric flask with chloroform. The absorbance was measured at 470 nm. Atropine was used to establish a standard curve (*y =* 0.1277*x* + − 0.0492, *R*^2^*=* 0.9923). The result was expressed as mg atropine equivalent (AE)/g of dry extract.

### Determination of antioxidant activity of the flower bud extracts

The antioxidant activity of the dried flower bud extracts was measured using a DPPH radical scavenging assay (Wijekoon et al., 2011). Shortly, 1 ml of each extract in methanol at various concentrations (0–150 μg/ml) was mixed with 1 ml of DPPH solution (25 μg/ml in methanol). After incubation at 37°C for 30 min, the absorbance of the mixture was measured at 517 nm. Absolute methanol and ascorbic acid were used as the negative control and positive control, respectively. The DPPH scavenging activity was calculated as follows:


$$\text{DPPH scavenging activity}[\%]=\frac{A_{1}{-A}_{2}}{A_{1}}\times 100$$ (1)

where *A*_1_ and *A*_2_ represent the absorbances of the negative control and the samples (extract or ascorbic acid), respectively.

### Determination of antibacterial activity of the flower bud extracts

The antibacterial activities of the dried flower bud extract against two *H. pylori* strains, ATCC 51932 and ATCC 43504, and *S. typhimurium* ATCC 13311, were evaluated using a microbroth dilution assay (Ngan et al., 2012). The extracts were diluted in DMSO (2.5%) to various concentrations ranging from 0 to 5 mg/ml. Each extract, 50 μl in volume, was dispensed into individual wells of a 96-well plate containing 50 μl of medium and 40 μl of the bacterial suspension (10^8^ CFU/ml). Negative and positive control wells received 50 μl of DMSO (2.5%) and 50 μl of amoxicillin (ranging from 0 to 0.1 μg/ml), respectively. The plates were then incubated for 24 h for *S. typhimurium* and 48 h for *H. pylori* at 37°C. Minimum inhibitory concentration (MIC) values were determined as the lowest concentration visibly inhibiting bacterial growth, with resazurin serving as an indicator.

### Determination of enzyme inhibitory activity of the flower bud extracts

Crude urease from *H. pylori* ATCC 43504 was extracted following the method outlined by Icatlo et al. (1998). The enzyme inhibition was determined using the methodology reported by Ngan et al. (2012) with minor modifications. Briefly, a urease solution was prepared by dissolving 0.25 mg of the crude urease (equivalent to 0.04 urease units) in 0.1 ml of EDTA-sodium phosphate buffer (pH 7.3). The dried flower bud extracts were then diluted in DMSO (2.5%) to concentrations ranging from 0 to 500 μg/ml. In a 96-well plate, 10 μl of each extract was mixed with 10 μl of the urease solution and 30 μl of EDTA-sodium phosphate buffer. After incubating at 37°C for 1 h, 50 μl of urea solution (0.24 mg/ml in EDTA-sodium phosphate buffer) was added to the mixture and incubated for an additional 30 min at 37°C. Blank and background wells were prepared containing DMSO (2.5%) and the extracts, respectively, with the enzyme solution inactivated by heating at 100°C for 30 min. Subsequently, stop solutions were added, consisting of 40 μl of solution A (40% sodium salicylate and 0.3% sodium nitroprusside) and 60 μl of solution B (0.5% sodium hydroxide and 0.042% sodium hypochlorite), to the reaction mixture. The ammonia released due to enzyme activity was quantified by measuring the absorbance at 625 nm using a Microlisa Plus microplate reader (Micro Lab Instruments, India). The protein content was determined using a Bradford protein assay kit (Thermo Fisher Scientific, Rockford, USA) with BSA as the protein standard. Thiourea served as a reference inhibitor. One urease unit is defined as the amount of enzyme that produces 1 mM NH_3_/min at 37°C and pH 7.3.

The α-glucosidase inhibition was determined following the method outlined by Shai et al. (2011) with slight modifications. Shortly, the reaction mixture was prepared by mixing 100 μl of potassium phosphate buffer (100 mM, pH 6.8), 20 μl of α-glucosidase (2.0 U/ml), and 40 μl of each extract (ranging from 0 to 500 μg/ml). This mixture was then incubated at 37°C for 15 min before adding 40 μl of pNPG (5 mM). After an additional incubation period of 20 min at 37°C, 100 μl of Na_2_CO_3_ (0.1 M) was added to stop the reaction. The absorbance of the resulting mixture was measured at 405 nm. Acarbose was employed as the positive control. The inhibitory activities of both urease and α-glucosidase were calculated as follows:


$$\text{Enzyme inhibitory activity}[\%]=\frac{\left(A_{c}-A_{s}\right)}{A_{c}}\times 100$$ (2)

where *A_c_* represents the absorbance of the negative control and *A_s_* represents the absorbance of the tested extracts.

### Statistical analysis

The MIC (minimum inhibitory concentration) values for each tested extract and amoxicillin were determined based on at least three independent experiments performed in triplicate (*n* ≥ 9). The dried flower bud extracts were categorized into groups with MIC values of ≤ 0.13, > 0.13 to < 0.63, 0.63–1.25, > 1.25 to < 2.5, and ≥ 2.5 mg/ml, indicating extremely high, high, moderate, low, and no inhibitory activity against the growth of tested bacteria, respectively (Ngan et al., 2021). All experiments, except for MIC determination, were performed in triplicate, and data were shown as mean ± standard deviations (*n* ≥ 3). Analysis of variance (ANOVA) using Tukey’s multiple comparison test (*P* < 0.05) was conducted using Minitab software (Minitab^®^ version 19.2020.1, Minitab LLC, State College, PA, USA). IC_50_ values were depicted using GraphPad Prism 10 software (San Diego, CA).

## Results

### Effect of solvents on extraction yield and total phytochemical content

The effect of different solvents on the yields of dried flower bud extracts of *C. operculatus* is shown in Table 1. Methanol yielded the highest extract (19.6%), followed by chloroform (12.0%), ethyl acetate (11.2%), butanol (10.1%), ethanol (10.1%), and water (9.9%), whereas hexane yielded the lowest extract (0.4%).

**Table 1:** Effect of solvents on extraction yield and total phytochemical content in the flower bud extracts of *C. operculatus*

Extract	Yield [%]	TPC	TFC	TTC	TSC	TAC
Hexane	0.4 ± 0.02 ^e^	85.4 ± 1.05 ^c^	81.1 ± 0.19 ^a^	32.7 ± 3.76 ^a^	154.2 ± 1.98 ^e^	1.0 ± 0.06 ^c^
Chloroform	12.0 ± 0.08 ^b^	59.1 ± 2.11 ^e^	43.2 ± 0.36 ^f^	21.9 ± 0.96 ^b^	287.2 ± 3.64 ^a^	1.2 ± 0.09 ^bc^
Ethyl acetate	11.2 ± 0.35 ^c^	65.0 ± 0.61 ^d^	44.2 ± 0.43 ^e^	28.0 ± 2.10 ^a^	252.6 ± 6.19 ^c^	1.6 ± 0.18 ^a^
Butanol	10.1 ± 0.10 ^d^	54.1 ± 2.65 ^f^	43.9 ± 0.26 ^ef^	14.1 ± 0.83 ^c^	270.8 ± 0.73 ^b^	1.0 ± 0.11 ^c^
Ethanol	10.1 ± 0.48 ^d^	101.5 ± 0.61 ^b^	47.5 ± 0.29 ^d^	11.6 ± 0.83 ^c^	245.4 ± 1.72 ^c^	1.2 ± 0.02 ^bc^
Methanol	19.6 ± 0.37 ^a^	87.8 ± 1.61 ^c^	48.5 ± 0.23 ^c^	3.0 ± 0.48 ^d^	163.6 ± 2.42 ^d^	1.1 ± 0.09 ^bc^
Water	9.9 ± 0.11 ^d^	328.9 ± 0.61 ^a^	75.8 ± 0.39 ^b^	16.3 ± 1.27 ^c^	8.3 ± 2.52 ^f^	1.4 ± 0.06 ^ab^

All data were expressed as mean ± standard deviation (*n* ≥ 3); different letters in the column indicate significant differences at *P* < 0.05 using one-way ANOVA by Tukey’s multiple comparison test; TPC stands for total phenolic content [mg GAE/g extract]; TFC stands for total flavonoid content [mg QE/g extract]; TTC stands for total tannin content [mg CE/g extract]; TSC stands for total triterpenoid saponin content [mg OAE/g extract]; TAC stands for total alkaloid content [mg AE/g extract]; GAE stands for gallic acid equivalent; QE stands for quercetin equivalent; CE stands for catechin equivalent; OAE stands for oleanolic acid equivalent; AE stands for atropine equivalent

The results of total phenolic, flavonoid, tannin, triterpenoid saponin, and alkaloid content (TPC, TFC, TTC, TSC, and TAC respectively) in the flower bud extracts (Table 1) indicated the presence of all tested phytochemicals in the solvent extracts. The water extract showed high levels of phenolics (TPC of 328.9 mg GAE/g extract) and flavonoids (TFC of 75.8 mg QE/g extract) but had significantly lower TTC and TSC. Conversely, the hexane extract had the highest TFC (81.1 mg QE/g extract) and TTC (32.7 mg CE/g extract) but lower TPC and TSC compared to other extracts. Throughout the experiment, TSC was mainly concentrated in the chloroform, butanol, ethyl acetate, and ethanol extracts (TSCs ranging from 245.4 to 287.2), followed by the methanol and hexane extracts (TSCs ranging from 154.2 to 163.6), while surprisingly, it was present at very low levels in the water extract. Moreover, in addition to containing abundant triterpenoid saponins, the methanol and ethanol extracts were rich in phenolics (87.8 and 101.5 mg GAE/g extract, respectively), had low TFC content, and contained minimal TTC. Among the phytochemicals present in all solvent extracts of the dried flower buds, alkaloids were found in the lowest amounts.

### Antioxidant activity of the flower bud extracts

**Fig. 1:**
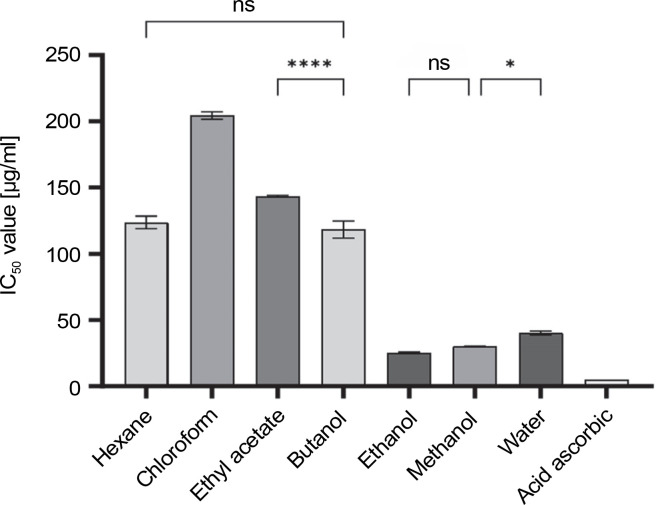
DPPH radical scavenging IC_50_ values of different *C. operculatus* flower bud extracts; ns – depicts no significant difference at *P >* 0.05, * indicates a significant difference at *P <* 0.05, and **** indicates a significant difference at *P <* 0.0001 using one-way ANOVA by Tukey’s multiple comparison test

The antioxidant activities of different solvent extracts are presented in Figure 1. These extracts exhibited significantly different antioxidant activity levels based on the solvents used during extraction. Among them, the ethanol extract exhibited the strongest antioxidant activity (IC_50_ values of 25.2 μg/ml), followed by the methanol and water extracts with moderate activity (IC_50_ of 30.3 and 40.2 μg/ml, respectively). Conversely, the butanol, hexane, and ethyl acetate extracts showed low to no antioxidant capacity (IC_50_ range of 118.4–143.3 μg/ml), and the chloroform extract exhibited the lowest activity (IC_50_ of 204.4 μg/ml). It is worth noting that all tested extracts showed lower antioxidant effects compared to ascorbic acid (IC_50_ of 5.2 μg/ml).

### Antibacterial activity of the flower bud extracts

The bacterial growth inhibitory activities of the flower bud extracts against two strains, *H. pylori* ATCC 51932 and ATCC 43504, and *S. typhimurium* ATCC 13311, are shown in Table 2. The antibacterial effectiveness of these extracts varied based on the extract type and concentration, as well as the specific bacteria tested.

**Table 2:** MIC values of different *C. operculatus* flower bud extracts against *H. pylori* and *S. typhimurium*

Extracts	MIC [mg/ml]		
	*H. pylori* ATCC 51932	*H. pylori* ATCC 43504	*S. typhimurium* ATCC 13311
Hexane	0.06	0.13	0.31
Chloroform	0.63	1.25	0.63
Ethyl acetate	1.25	1.25	0.63
Butanol	5.0	2.5	1.25
Ethanol	0.63	0.63	0.63
Methanol	0.63	1.25	1.25
Water	2.5	5.0	0.63
Amoxicillin [μg/ml]	0.01	0.01	0.03

MIC stands for minimum inhibitory concentration; MIC values of each tested extract and amoxicillin were obtained from at least three independent experiments performed in triplicate (*n* ≥ 9); tested extracts with MIC values of ≤ 0.13, > 0.13 to < 0.63, 0.63–1.25, > 1.25 to < 2.5, and ≥ 2.5 mg/ml were respectively classified as extremely high, high, moderate, low, and no inhibitory activity against the growth of tested bacteria

The hexane extract showed extremely high antibacterial activity against both strains of *H. pylori* ATCC 51932 and ATCC 43504 (MIC values of 0.06 and 0.13 mg/ml, respectively), followed by moderate activity from the ethanol, methanol, chloroform, and ethyl acetate extracts (MIC values of 0.63–1.25 mg/ml). However, the water and butanol extracts showed no effect on the strains’ growth.

The hexane extract also exhibited a high inhibitory effect on the growth of *S. typhimurium* ATCC 13311, with a MIC of 0.31 mg/ml, significantly lower than its effect on *H. pylori*. Additionally, all other extracts induced moderate antibacterial activity against *S. typhimurium*, with MIC values ranging from 0.63 to 1.25 mg/ml.

The MIC values of amoxicillin against both strains of *H. pylori*, ATCC 51932 and ATCC 43504 (0.01 μg/ml), indicated that these strains were susceptible to amoxicillin because the MIC resistance breakpoints for amoxicillin were reported to be > 0.5 μg/ml (Garciá-Arata et al., 1999; Kim et al., 2011). According to EUCAST (2015), *Salmonella* spp. which showed a MIC value of amoxicillin of ≤ 8.0 μg/ml is considered susceptible. This indicated that the *S. typhimurium* strain ATCC 13311 was also susceptible to amoxicillin, with an MIC of 0.03 μg/ml.

### Enzyme inhibitory activity of the flower bud extracts

The enzyme inhibition activities of dried flower bud extract from *C. operculatus* against *H. pylori-*urease and α-glucosidase varied significantly among the extracts (Table 3). The hexane extract displayed the most effective inhibitory activity on urease (IC_50_ value of 4.5 μg/ml), followed by the ethanol, chloroform, and ethyl acetate extracts (IC_50_ values of 8.5–9.6 μg/ml), and then the methanol, butanol, and water extracts (IC_50_ values of 12.2–13.8 μg/ml). All these extracts demonstrated significantly better urease inhibition than the standard thiourea (IC_50_ of 44.3 μg/ml). In contrast, the hexane and ethyl acetate extracts showed no activity against α-glucosidase (IC_50_ > 200 μg/ml), while the ethanol, butanol, and chloroform extracts exhibited weak inhibitory effects (IC_50_ values of 90.6–150.6 μg/ml). The water and methanol extracts showed potent inhibitory effects on the enzyme (IC_50_ values of 9.9 and 15.1 μg/ml, respectively), with both extracts presenting stronger activity compared to the positive compound acarbose (IC_50_ value of 74.9 μg/ml).

**Table 3:** Inhibitory effect of different *C. operculatus* flower bud extracts on *H. pylori*-urease and α-glucosidase activities

Extracts	IC_50_ [μg/ml]	
	*H. pylori*-urease	α-Glucosidase
Hexane	4.5 ± 0.17 ^e^	268.2 ± 18.61 ^a^
Chloroform	8.5 ± 0.15 ^d^	150.6 ± 10.60 ^c^
Ethyl acetate	9.6 ± 0.18 ^d^	203.7 ± 12.35 ^b^
Butanol	12.2 ± 0.07 ^c^	101.3 ± 0.48 ^d^
Ethanol	8.8 ± 0.07 ^d^	90.6 ± 3.69 ^de^
Methanol	12.3 ± 0.20 ^c^	15.1 ± 0.30 ^f^
Water	13.8 ± 0.27 ^b^	9.9 ± 1.33 ^f^
Thiourea	44.3 ± 1.12 ^a^	ND
Acarbose	ND	74.9 ± 3.56 ^e^

All data were expressed as mean ± standard deviation (*n ≥* 3); different letters in the column indicate significant differences at *P* < 0.05 using one-way ANOVA by Tukey’s multiple comparison test; ND stands for not determined

## Discussion

There is an increasing interest in using natural compounds as functional foods for treating various diseases and promoting human health (Wijekoon et al., 2011; Seo et al., 2014; Uysal et al., 2021). This study showed that the phytochemical constituents and content of these bioactive compounds vary significantly and depend on the solvent used. High extraction yields were achieved using methanol, chloroform, ethyl acetate, and ethanol, whereas hexane resulted in the lowest extraction yield. This indicates that *C. operculatus* flower buds contain a wider range of bioactive compounds with higher polar properties compared to those with low or nonpolarity. Similarly to previous studies on flower bud extracts from *C. operculatus*, the TPC was higher in aqueous, ethanol, and methanol extracts than in other organic extracts (Wijekoon et al., 2011; Rebaya et al., 2015; Temesgen et al., 2022). The phytochemicals isolated and identified from leaves and flower buds of *C. operculatus* were well described and classified into three major groups: oleanane and ursane triterpenoids, C-methylated flavonoids, and polycyclic phloroglucinols (Pham et al., 2020). Among the flavonoids in the flower buds, chalcones were reported as the major bioactive constituents mainly found in nonpolar and low-polar solvent extracts such as petroleum ether, hexane, and ethyl acetate (Ye et al., 2004; Kumar and Pandey, 2013). Especially, DMC, a major flavonoid, is known for its health benefits, including potent antidiabetic and antiobesity activities (Mai et al., 2010; Hu et al., 2014). Saponins are triterpenoid and steroid glycosides naturally occurring in many medicinal plants and are considered potential pharmaceutical and/or nutraceutical agents (Juang and Liang, 2020; Pham et al., 2020; Elouafy et al., 2023). The pentacyclic oleanane, ursane, lupane, and tetracyclic dammarane are the four major skeletons of triterpenoid saponins (Juang and Liang, 2020). In our research, all flower bud extracts of *C. operculatus* were rich in triterpenoid saponins, except for the water extract, similar to the previous study on *Juglans regia* leaf extracts (Elouafy et al., 2023). Oleanane and ursane triterpenoids were predominantly found as the main components of the leaves, with lupane triterpenoids also isolated from the flower buds of *C. operculatus* (Pham et al., 2020). Oleanane saponins have garnered significant interest for their antitumor, antiviral, and immunomodulatory effects (Juang and Liang, 2020), whereas tannins and alkaloids were determined to be present in the flower buds but at significantly lower levels compared to other components. Furthermore, the chemical composition and biological activity of tannins and alkaloids in *C. operculatus* have not been reported.

Oxidative processes result in the damage of numerous macromolecules, such as proteins, lipids, and DNA, consequently causing the progression of various diseases (Uysal et al., 2021). In recent years, natural antioxidants have garnered increasing attention as they could play various roles such as metal chelators, reducing agents, hydrogen donors, and free radical quenchers to prevent damage caused by free radical-induced oxidative stress (Ye et al., 2013). Among the extracts tested in the current study, ethanol, methanol, and water extracts exhibited the highest antioxidant activity, with IC_50_ values of 25.2, 30.32, and 40.2 μg/ml, respectively. Notably, the ethanol and methanol extracts exerted significantly stronger effects than the ethanol extract of *C. operculatus* flower buds (IC_50_ of 39.3 μg/ml) reported by Dung et al. (2008) and the methanol extracts of *Syzygium aromaticum* flower buds (303.6 μg/ml) (Temesgen et al., 2022) and *Rosmarinus officinalis* (532.0 μg/ml) (Nguyen et al., 2021). This result indicates that the bioactive constituents present in *C. operculatus* flower buds possess potent antioxidant activity and therefore could scavenge diverse reactive oxygen species, including hydroxyl radicals, to protect the human body against oxidative damage (Ye et al., 2013; Nguyen et al., 2021).

Chronic infection of *H. pylori* in humans leads to gastritis, duodenal peptic ulcer disease, and gastric cancer (Wroblewski et al., 2010). Currently, eradication of *H. pylori* relies heavily on multidrug therapies, and the emergence of *H. pylori* strains resistant to multiple antibiotics and their reinfections have reduced the effectiveness of the therapy (Wroblewski et al., 2010; Elbehiry et al., 2023). Therefore, plant-based therapies for the prevention and treatment of bacterial infections are increasingly preferred. In our previous study, the ethanol extract from leaves of *C. operculatus* was shown to be one of the most effective anti-*H. pylori* materials against ATCC 51932 and three other clinical strains (MICs ranging from 0.31 to 0.97 mg/ml) (Ngan et al., 2017). The present study revealed that the hexane extract from the flower buds of *C. operculatus*, which is rich in flavonoids and phenolics, also exhibited a potent growth inhibitory effect (MIC of 0.06–0.13 mg/ml) on two *H. pylori* strains, ATCC 51932 and ATCC 43504. Additionally, it was reported that the hexane fraction (MICs of 0.75–1.0 mg/ml) and ethyl acetate fraction (MICs of 0.2–0.25 mg/ml) derived from the red flower ethanolic extract of *Hibiscus rosa sinensis*, along with several flavonoid and phenolic compounds, showed the most growth inhibitory activity against clinical isolates of *H. pylori* (Hieu et al., 2020; Ngan et al., 2021). The anti-*H. pylori* activity of *C. operculatus* flower bud extracts could be more effective than those of other medicinal plants such as *Cinnamomum zeylanicum* (MICs of 1.25–5.0 mg/ml), *Cichorium intybus* (MICs of 1.25–10 mg/ml), and *Foeniculum vulgare* (MIC of > 10 mg/ml) (Nostro et al., 2005).

*C. operculatus*, with its potential medicinal properties, has traditionally been used to prevent foodborne illnesses caused by common bacteria, including *Salmonella* infections. *S. enterica Typhimurium* and *Enteritidis* are known to be the most important strains infecting humans, especially in tropical communities (Jajere, 2019). In previous research, essential oil from *C. operculatus* flower buds has demonstrated antimicrobial activity against nine foodborne pathogens, including *S. typhimurium* and *S. enteritidis*, with a MIC of 0.004 mg/ml. However, the ethanolic extract of the flower buds showed no inhibition against these bacterial pathogens (MIC > 32 mg/ml) (Dung et al. 2008). Our present study found that the flower bud extracts exhibited anti-*S. typhimurium* activity at the tested concentrations, particularly the hexane extract, which displayed potent antibacterial activity with a MIC of 0.31 mg/ml. In addition, the essential oil and solvent extracts from *C. operculatus* flower buds have demonstrated strong antibacterial activity against several strains of plant pathogenic bacteria, *Xanthomonas* spp. (MICs of 0.031–0.125 and 0.25–0.5 mg/ml, respectively) (Bajpai et al., 2010). These results suggest that extracts isolated from *C. operculatus* flower buds could serve as promising antibacterial agents against *H. pylori* and *Salmonella* infections.

The inhibitory activity of the extracts from *C. operculatus* flower buds against *H. pylori* urease, especially the hexane extract, was initially observed in our study. This urease inhibitory effect may be attributed to the antibacterial property of this extract on bacterial growth. Urease is closely related to the bacterial ability to survive and persistently colonize in the hostile conditions of the human stomach (Kusters et al., 2006). Therefore, inhibition of urease activity is a key measure in preventing *H. pylori* infections. A previous study reported that acetone extracts of *Fagonia arabica* and methanol extracts of *Casuarina equisetifolia* could also inhibit *H. pylori* urease activity (Amin et al., 2013). A recent study on the flower of *H. rosa sinensis* indicated that ethanol extract and its water and ethyl acetate fractions also showed inhibitory effects on *H. pylori* urease, whereas its hexane fraction had no activity (Ngan et al., 2021).

In recent years, it has been reported that *H. pylori* infections could enhance the incidence of diabetic complications, particularly type 2 diabetes (Mansori et al., 2020). In addition to its antibacterial activity, flavonoid and polyphenol-rich extracts of *C. operculatus* are also believed to have clinical applications in preventing obesity and type II diabetes (Mai and Chuyen, 2007; Du et al., 2022) due to their potent inhibitory effects on pancreatic lipase, α-amylase, and α-glucosidase. Previously, it was shown that the aqueous extract of *C. operculatus* flower buds possessed a higher inhibitory effect on α-glucosidase compared to other plants such as *Camellia sinensis*, *Psidium guajava*, *Nelumbo nucifera*, and *Sophora japonica* (Mai and Chuyen, 2007). In our results, the water and methanol extracts displayed potent α-glucosidase inhibitory effects and were significantly better than both the positive compound acarbose and the ethanol extract. Farrerol 7-O-β-d-(6-O-galloyl) glucopyranoside, a C-methyl flavonoid isolated from the ethanol extract of *Cleistocalyx conspersipunctatus* leaves, also displayed potent α-glucosidase inhibitory activity, surpassing that of a known inhibitor, corosolic acid (Du et al., 2022).

## Conclusion

The biological activities of different extracts from *C. operculatus* flower buds are attributed to their phytochemical compositions. The study results indicated that ethanol, methanol, and water extracts could serve as potent sources of antioxidants. The hexane extract exhibited the highest antibacterial activity against *H. pylori* and *S. typhimurium*. The active ingredients in the hexane extract may inhibit the growth of *H. pylori* by inhibiting urease activity. Additionally, the methanol and water extracts were found to possess inhibitory effects on α-glucosidase. These extracts may provide a possible approach for food and pharmaceutical applications.
